# Construction and optimization of Green Infrastructure Network in mountainous cities: a case study of Fuzhou, China

**DOI:** 10.1038/s41598-024-57567-0

**Published:** 2024-05-24

**Authors:** He Huang, Danling Fu, Guochang Ding, Chen Yan, Xiangcai Xie, Yaling Gao, Qunyue Liu

**Affiliations:** 1 College of Landscape Architecture and Art, Fujian Agriculture and Forestry University, Fuzhou, 350002 China; 2 National Center for Water Conservancy Scenic Area Research, Fuzhou, 350002 China; 3 Fujian University of Technology, Fuzhou, 350118 China

**Keywords:** Green infrastructure network, Mountainous cities, Circuit theory, Morphological spatial pattern analysis, Ecology, Environmental sciences

## Abstract

Green infrastructure networks enhance the protection and improvement of urban ecological environments, augment the efficiency and quality of ecosystem services, and furnish residents with healthier and more comfortable living conditions. Although previous research has investigated the construction or optimization methods of green infrastructure networks, these studies have been relatively isolated and lacking in case studies for mountainous cities. In the development of green infrastructure, mountainous cities must specifically consider the impact of terrain on network construction. Taking Fuzhou, a mountainous city in China, as an example, this study constructs and optimizes the green infrastructure network by employing morphological spatial pattern analysis, connectivity analysis, the Minimum Cumulative Resistance model, and circuit theory. These methodologies increase the connectivity of the Green Infrastructure within the study area, thereby promoting the health of the local ecosystem and creating conducive circumstances for the city’s sustainable development. The findings reveal that: (1) Green infrastructure in Fuzhou takes up 5366.38 ha, constituting 21.76% of the study area, primarily situated in the northwest and south; (2) Fuzhou’s Green Infrastructure network comprises 10 hubs and 17 corridors with a hub area of 1306.98 ha, predominantly distributed in the mountains encircling the city, including Meifeng Mountain, Gaogai Mountain, and Qingliang Mountain; (3) Based on optimization, the circuit centrality index categorizes hub importance into three protection levels, pinpointing nine crucial protected areas in the corridors and 680 areas requiring enhancement, including 68 areas for first-level improvement, 149 areas for second-level improvement, and 463 areas for third-level improvement. This research offers a methodological reference for constructing and optimizing green infrastructure networks in mountainous cities, providing both theoretical and practical foundations for optimizing green infrastructure networks in Fuzhou City.

## Introduction

Urban development and construction have engendered numerous ecological issues in recent decades, including global warming, biodiversity loss, and haze pollution. These problems have become a shared concern and pressing matter for governments and scholars worldwide^1–3^. Consequently, to mitigate these environmental challenges, the concept and theoretical methods of green infrastructure (GI) networks were introduced in the 1990s^4^, offering crucial support and planning guidance for urban ecological development. Following years of research and practice, scholars have achieved a more unified understanding of the GI network’s connotation^5^, which is regarded as a network system comprising intertwined natural and artificial green landscape spaces^6^. GI networks can ameliorate habitat fragmentation, enhance habitat quality, and ensure the ecological processes of natural green spaces^7,8^, ultimately achieving the objectives of ecosystem health, biodiversity, and ecological resource protection. In researching the ecological functions of GI, scholars have conducted extensive studies, indicating that GI possesses ecological functions in climate regulation^9–12^, air regulation^13^, hydrological regulation^14–16^, human health^17,18^, and other aspects. Additionally, they believe that GI networks can effectively enhance the efficiency of ecosystem services^19^.

**Figure 1:**
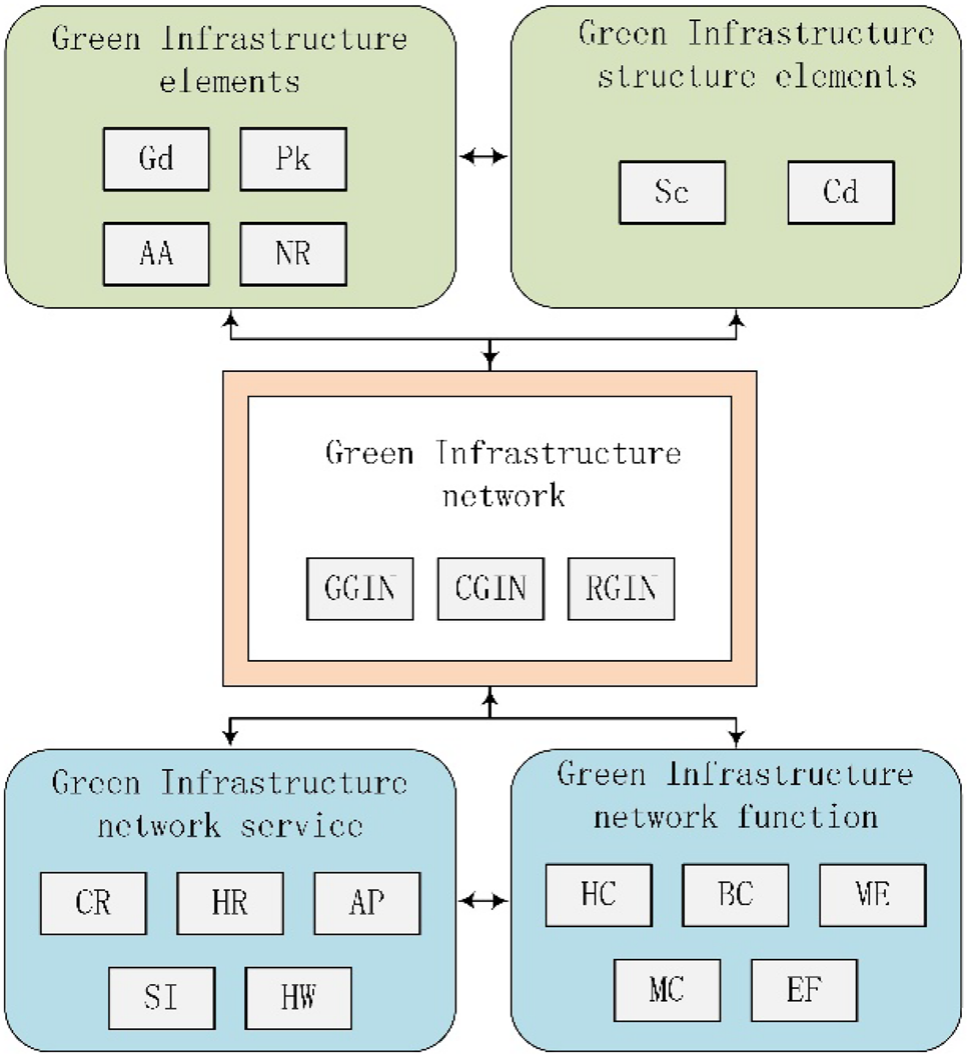
Framework of natural elements, spatial structure, ecological services, and ecological functions of green infrastructure network. The framework is divided into three parts: the upper part represents the GI network's natural elements and structural components, the middle part depicts the GI network and spatial classification, and the lower part illustrates the GI network's ecological services and functions. The interrelationships between the three parts are indicated by arrows. Keywords: Gd: Gardens; Pk: Parks; AA: Aquatic area; NR: nature reserve; Sc: Sources; Cd: Corridors; GGIN: Garden-level GI network; CGIN: Community-level GI network; RGIN: Regional-scale GI network; CR: Climate regulation; HR: Hydrological regulation; AP: Air quality regulation; SI: Soil improvement; HW: Promotion of human health and well-being; HC: Habitat conservation; BC: Biodiversity conservation; ME: Maintenance of ecological processes; MC: Material cycling; EF: Energy flow.

In terms of spatial composition, GI networks primarily consist of hubs and corridors^20^. Hubs, the primary constituent elements of GI, serve as the main source of habitat and ecosystem services provision^19^. Although corridors connecting hubs offer limited ecosystem services, they are vital components of GI that facilitate connectivity, species migration, information exchange, and energy exchange, playing a significant role in preventing habitat fragmentation and maintaining ecological security patterns^20^. Spatially, GI can be categorized into garden-level, community-level, and city-level^21^. Garden-level GI constitutes the smallest unit, which might encompass a garden or pond. Community-level GI has a larger spatial scale, featuring corridors that connect multiple garden-level GI and more diverse ecological landscapes, such as communities and streets. City-level GI comprises multiple community-level GI connected by corridors, incorporating large-scale urban green spaces like parks and nature reserves and playing a critical role in supporting the entire urban ecology. Whether garden-level, community-level, or city-level GI networks, they are all open networks, signifying that garden-level GI can connect to other GI networks, and distinct city-level GI networks can interconnect through corridors. Additionally, GI possesses ecosystem service functions. Figure 1 depicts the concept, structure, and functional framework of the GI network.

Given the manifold benefits of GI networks in optimizing landscape patterns and promoting ecosystem health and service provision, scholars have extensively explored how to plan and optimize GI networks^10,20^. A relatively consistent paradigm, namely "Hubs-resistance surface-corridors", has emerged in the construction of GI networks^22^. The process of GI network construction and optimization typically involves three steps: (1) Identification of Hubs. Early studies on the identification of Hubs were somewhat subjective, typically selecting large green spaces or significant nature reserves. This approach favored the higher ecological quality of Hubs but neglected their supportive role in the spatial pattern of GI networks^23,24^. In recent years, researchers have progressively utilized the Methodology Morphological Spatial Pattern Analysis (MSPA) for its efficient retrieval of spatial data, a process streamlined by employing basic binary images. This approach has gained significant recognition as the primary means for spatial assessment and network establishment in the realm of GI. While this technique does indeed recognize the supportive function of the GI network structure in architectural terms, it regrettably overlooks the inherent ecological excellence of these Hubs which encompasses pivotal aspects like species diversity, vegetation coverage, and ecological suitability^25^. (2) Resistance Surface Construction and Corridors Extraction. The classic method for corridor extraction is the Minimum Cumulative Resistance (MCR) model, In contrast to traditional graph theory methods, the MCR model not only quantifies the degree of isolation between hubs but also takes landscape processes into account^22,25^. However, MCR necessitates assigning resistance values to each landscape grid in the study area, forming a resistance surface. Resistance values are typically assigned based on land use types without considering spatial differences, such as topography. Moreover, MCR exhibits a high degree of subjectivity, significantly affecting corridor selection^26^. (3) Evaluation and optimization of GI networks, network analysis methods based on graph theory are prevalent^27,28^. These methods employ α, β, and γ indices to assess the structural connectivity of the network, quantifying the number of hubs and corridors and their proportional relationships to evaluate network connectivity; however, they do not account for ecological processes. Some researchers have added codes to GI corridors to enhance their stability and achieve GI network optimization to some extent, but they have not considered the specific ecological conditions of existing corridors and Hubs^29^.

To address the current research gap, this study has refined the methods for identifying and optimizing GI networks, with several key advantages. Firstly, in selecting hubs, a combination of area, connectivity, and the intrinsic quality of ecological land is assessed, integrating scientific quantification with practical investigation. This approach considers both the spatial structure of GI networks and ensures the ecological quality of hubs, resulting in a more scientifically grounded and practical selection process. Secondly, the adoption of the MCR model for corridor extraction is advantageous because it incorporates both actual distance factors and ecological isolation, thereby comprehensively addressing ecological processes. Thirdly, the optimization of landscape resistance surface construction involves the correction of terrain factors, thereby fully considering their impact and resulting in a more rational identification of corridors^30^. Fourthly, this study employs circuit theory to optimize GI networks, conceptualizing electric charges as ecological flows within complex landscapes. Resistance in the circuit represents the resistance to ecological flow diffusion within landscape grids, with resistance values typically derived from the MCR resistance surface. This method effectively considers ecological processes^31^. By utilizing circuit theory to identify key areas within GI networks based on node centrality and the concept of "pinch points" and "obstacle zones," the study provides clear and precise optimization recommendations. Lastly, while previous research has predominantly focused on plain cities, high-density urban areas, suburbs, and coastal cities, there has been limited exploration of how to construct GI networks in mountainous urban areas. This study aims to address this gap by exploring the construction of GI networks in densely populated mountainous urban areas, thereby mitigating land use conflicts inherent to such terrain characteristics.

China is a country with abundant mountains, and numerous mountainous cities contribute significantly to its economic development. These cities exhibit not only economic prosperity, high population density, and elevated urbanization levels but also an abundance of mountains, hills, wetlands, and water systems in terms of ecological geography. Examples include Fuzhou, Xiamen, Hangzhou, Hong Kong, and Taipei, where natural Greenland offers vital resources and ecological barriers for urban areas^32^. However, with the acceleration of urbanization and sustained economic growth, ecological degradation, ecological land encroachment, habitat fragmentation, and other issues have sharply intensified, making ecological protection and urban development increasingly critical^33^. Fuzhou, a typical mountain city, has experienced rapid development in recent years, which has intensified the conflict between Greenland use and urban construction. Undoubtedly, the municipal government, while focusing on economic development, has also intensified its efforts and investments in ecological construction, such as afforestation, ecological urban and forest city construction, river ecological management, and other GI projects. Despite efforts, the government is yet to fully implement the integrated development of the GI spatial configuration. The fragmentation and isolation of ecological land impede the efficacy of GI’s environmental services and contributes to the lack of stability in the ecosystem. Moreover, urban habitat fragmentation also results in diminished species diversity and an increase in plant and pest-related ecological issues. Without comprehensive analysis and evaluation of the GI spatial configuration, mere augmentation of green spaces may prove inadequate for a land-constrained mountain city like Fuzhou. These approaches could impede the efficient use of scarce land resources, presenting obstacles to the city’s sustainable development^12^. Therefore, the application of spatial analysis in comprehensive GI network planning holds considerable significance for the development and environmental conservation in mountainous urban areas. This paper examines Fuzhou City in China as a case study and, based on the actual situation of a mountainous city, employs methods such as MSPA, core area index, connectivity analysis, MCR model, and circuit theory to identify and optimize the GI network in the research area. This paper constructs a Fuzhou GI network identification and optimization scheme based on multiple methods, providing a solution to improve Greenland use efficiency and achieve sustainable urban development in Fuzhou City. Simultaneously, it offers targeted policy recommendations and practical experience for government departments, enterprises, and researchers in ecological planning, land use, environmental protection, and other fields in other mountainous cities.

## Materials and methods

### Study area

**Figure 2:**
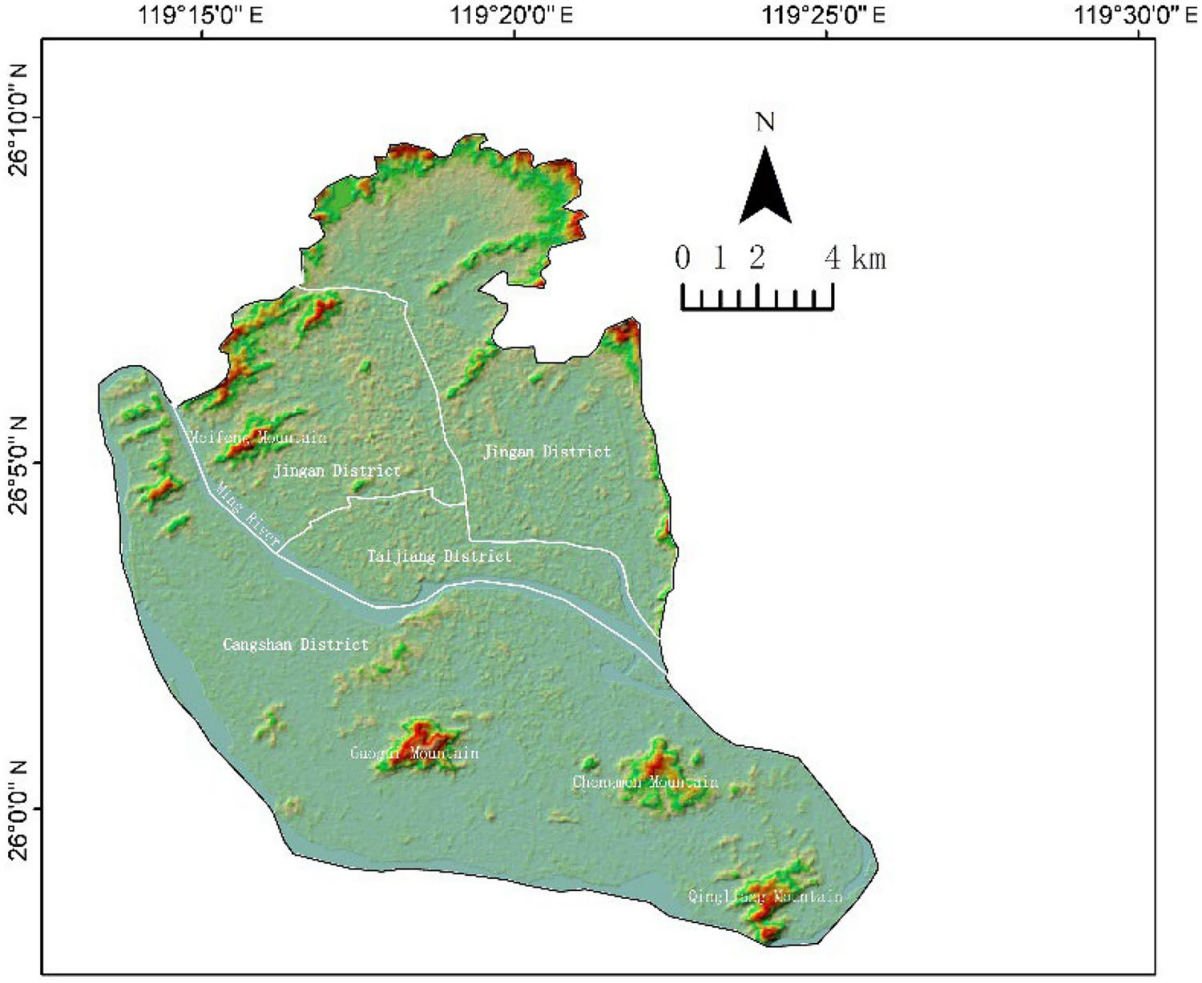
Map of Mountainous Terrain and Urban Blocks in the Study Area. Map created in ArcGIS 10.2, DEM Data was obtained from a mirror website of the Computer Network Information Center for Geospatial Data.

Mountainous terrain offers a diverse range of habitats for species, and the land in mountainous urban areas has increasingly been impacted and encroached upon by human activities. The ecological system of mountainous urban areas is more fragile than that of plain areas. Fuzhou (26° 4ʹ 28″ N, 119° 17ʹ 47″ E), situated on the southeast coast and adjacent to the Taiwan Strait, serves as the primary research area for this study. It encompasses five county-level administrative units (Fig. 2): Gulou District, Taijiang District, Jin’an District, Cangshan District, and Mawei District, with a total area of 24,665 ha and an average urbanization rate of 97.1% (Fuzhou Statistical Yearbook 2022). The area has a total population of 3.305 million, according to the 7th national census in 2020. The geographic environment is characterized by “pillow shaped mountains, river hugging, and sea-facing” features, with numerous hills and water systems. Over ten hills, such as Meifeng Mountain, Qingliang mountain, Gaogai mountain, and Chengmen Mountain, are present, and the largest water system is the Minjiang River, which flows through the main urban area and meets the Taiwan Strait at Fuzhou Langqi. The city is also crisscrossed by interconnected internal river networks. Fuzhou has a subtropical marine monsoon climate, with abundant rainfall, ample sunshine, and a warm and humid climate year-round, yielding rich natural resources. However, due to rapid economic development in recent years, conflicts between economic development and ecological protection have become increasingly severe. Consequently, this study focuses on the main urban area of Fuzhou and identifies Greenland, such as forest land and farmland, as GI, since green areas and water bodies exhibit significant differences in ecological systems. This study identifies network hubs and corridors through the analysis of GI elements and distribution patterns and constructs GI networks. The study optimizes the networks using GI network planning methods to enhance the GI health and service capabilities of Fuzhou City and to provide suggestions and practical references for ecological construction in economically developed mountainous urban areas.

The research area data was downloaded from the Geographic Spatial Data Cloud website in 2021 (https://www.gscloud.cn/home#page1), including Landsat8 ETM remote sensing images and DEM elevation data with a spatial resolution of 30 m. ENVI 5.1 and ArcGIS 10.7 software were employed to preprocess the ETM image through radiometric calibration, atmospheric correction, multispectral fusion, and cropping. Supervised classification and visual interpretation methods were used to obtain the land-use/land-cover map, which categorized land cover into five groups: construction land, water bodies, forest land, bare land, and farmland.

### Methods

**Figure 3:**
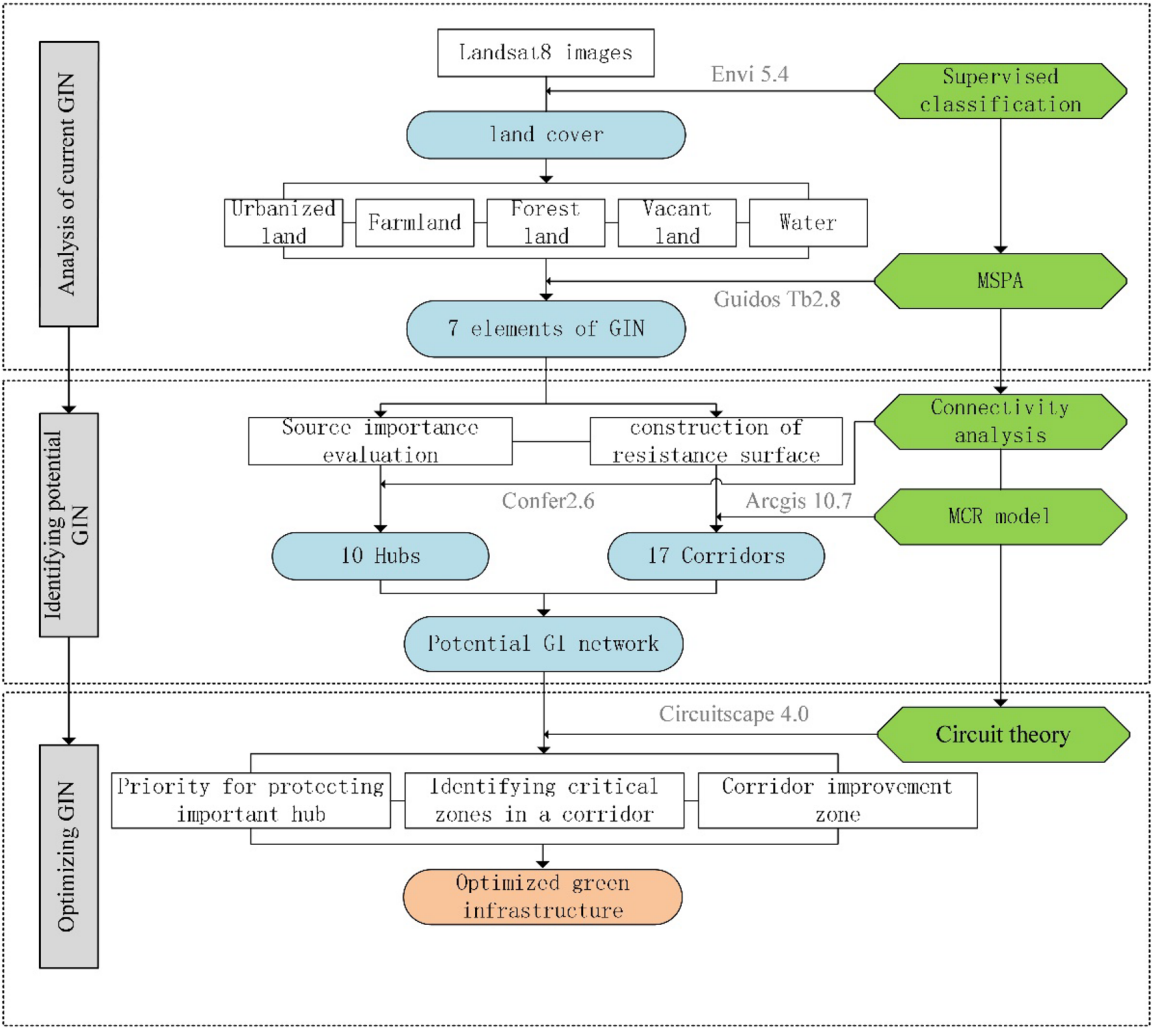
Research flowchart. Map created in Microsoft Visio.

The aim of this case study is to investigate the current state of the GI network elements and distribution in the study area, identify the GI network, and optimize it to form a comprehensive GI network ecosystem, offering a reference for ecological planning and construction in economically developed mountainous urban areas. The technical process of this study was divided into three steps (Fig. 3): (1) MSPA was employed to comprehend the current pattern of the GI network in the study area and identify the GI network elements. (2) The GI network identification in the study area was completed by extracting the source site and corridor. The source site selection was based on the core area and connectivity index to identify the urban GI network source site. Optimal corridor identification was achieved by constructing a terrain-modified resistance surface and utilizing the MCR model. The identified source site and corridor constitute the GI network in the study area, but the network is incomplete and requires further optimization. (3) Utilizing the backing of circuit theory, the analysis of networks is conducted using the sophisticated Circuitscape 4.0 software. This analysis discerns the significant sectors, pivotal zones, and vulnerable sectors within the GI network. Subsequently, the GI network is refined and enhanced through optimization..

#### Analysis of GI pattern based on MSPA

MSPA is a morphology-based spatial pattern analysis method that segments binary images based on the size, shape, and spatial distribution of pixels, forming seven types of spatially meaningful indicators, such as core, islet, loop, bridge, perforation, edge, and branch^34^ (see Table 1). The types, quantity, and spatial patterns are represented through illustration and digitalization. In this study, the Guidos Toolbox 2.8 platform was utilized for MSPA analysis. The process includes: (1) preparation of binary images by setting forest and farmland as GI and other land-use types as the background color using GIS software to export GeoTif format binary images with a raster size of 30 m; (2) parameter setting, taking into account the complexity of urban landscapes and previous research results^22^, the neighborhood rule was set to 8, and the edge width was set to 1 (30 m); (3) MSPA processing, using the Pattern module in Guidos to process the binary images and obtain the corresponding MSPA landscape type GeoTif image.

**Table 1:** The delineation of landscape categories in MSPA and their corresponding representation of urban green infrastructure components.

Landscape classes	Ecological meaning
Core	The core region of GI, encompassing peri-urban forest reserves, natural sanctuaries, and expansive urban parks with abundant vegetative cover, serves as vital terrain for safeguarding biodiversity and upholding natural ecological processes
Islet	Isolated patches of green space, lacking efficient connectivity with the external environment, such as residential green areas within urban settings, community parks, and small-scale parkland
Bridge	Linear green spaces, serving as conduits for the exchange and circulation of green energy between core regions of GI, are known as “green belts.” The greater their number, the enhanced the connectivity between core regions becomes
Loop	Efficient pathways for energy exchange and circulation within the core regions of GI
Edge	The green belt at the intersection of the core region of GI and the non-GI areas typically encompasses the peripheral forested areas of conservation zones or large parks
Perforation	Within the heart of the GI core region, there exists an inner expanse of vegetated zones that have suffered degradation due to both anthropogenic and natural disturbances
Branch	Severed corridors in regions significantly impacted by anthropogenic disruptions

#### Identification of GI network components

##### Hub identification methods

The GI network hubs are typically chosen from the more significant ecological source areas within the region. According to island biogeography theory, the quality of source areas is determined by factors such as patch area, connectivity, species diversity, and others^20^. Historically, the identification of key source areas was predominantly guided by landscape pattern indices or size considerations. Although landscape pattern indices include spatial structural benefits^23^, they neglected the inherent quality of the source areas and the biomass they can offer. There is a positive correlation between the area of Greenland within source areas and the amount of biomass, thus, depending solely on one aspect could result in a biased view. Therefore, this research extends its approach beyond using connectivity indices that represent landscape pattern quality, by also considering area to reflect the inherent quality of ecological land. This approach subsequently provides the foundation for assessing the relevancy of source areas. The study employs core area (A), possible connectivity index (PC), and overall connectivity index (IIC) to describe the importance of patches (dI). The importance of patches is classified into three levels: extremely important, important, and general, and more critical source areas are selected as GI network hubs. The connectivity index is calculated using Confer 2.6 software, and the formula for the index calculation is^35^:

1 $$PC=\frac{\sum_{i=1}^{n}\sum_{j=1}^{n}{a}_{i}{a}_{j}{p}_{ij}^{*}}{{A}_{L}^{2}}$$

2 $$IIC=\frac{\sum_{i=1}^{n}\sum_{j=1}^{n}\frac{{a}_{i}{a}_{j}}{1+n{l}_{ij}}}{{A}_{l}^{2}}$$

3 $$dI\left(\%\right)=100\times \frac{I-{I}_{remove}}{I}$$

where n represents the total number of patches in the landscape, a_i_ and a_j_ are the contribution values of patch i and patch j, nl_ij_ refers to the number of connections between patch i and j, and p^*^_ij_ is the maximum possible spread of species between i and j. I represent the landscape connectivity index value, and I_remove_ represents the landscape connectivity value after removing a patch.

Additionally, the connectivity index results vary depending on the distance threshold setting in Confer. This study used nine different thresholds (500, 1000, 1500, 2000, 3000, 4000, 5000, 6000, 7000 m) and selected six representative patches for experimentation. The threshold value was determined based on the trend and stability of the connectivity changes (dPC and dIIC)^36^.

##### Identification of potential corridors

Construction of landscape resistance surface: During the process of species migration and diffusion, various resistances are encountered, and the strength of these resistances is determined by the obstructive effects of different landscapes on the diffusion of species. Both the spatial pattern of the landscape and the ecological quality can affect this ecological process. In this study, the MSPA analysis results were utilized as landscape pattern factors, while land-use type was employed as ecological quality factors. Expert consultation was used to score each factor. Furthermore, the influence of terrain on species migration was considered. The greater the slope, the greater the resistance; thus, the slope was used to modify the basic resistance surface, which was formulated as follows^29^:

4 $${R}_{j}^{\prime}={R}_{j}\times (1+i)$$

where R_j_ represents the modified resistance value of patch j, R_j_ represents the basic resistance value of patch j, and i is the slope value of patch j (in percentage).

Method for identifying corridors: The Minimal Cumulative Resistance Model (MCR) is employed to simulate the optimal path for ecological flow diffusion. This method assumes that a species overcomes the resistance between a certain source and other destinations (the resistance values are extracted from the above-mentioned resistance surface), identifies the line between the two sources with the minimum cumulative resistance, and regards it as the best migration route. By delineating ecological source areas and integrating cost distance and cost path functions within ArcGIS, all potential ecological corridors can be identified and extracted. This method is widely used in the field of regional conservation planning, and its formula is^25^:

5 $$MCR=min\sum_{j=n}^{i=m}({D}_{ij}\times {R}_{j})$$

where MCR represents the minimum cumulative resistance value that a species needs to overcome from the source to the target. i and j are different grid units, and D_ij_ represents the unit distance from the hub unit i to the hub unit j. R_j_ is the weighted resistance value of the j grid to the ecological flow.

#### Evaluation and optimization of GI network

This study employs circuit theory methods to optimize the GI network, quantifying node centrality, "pinch points," and "obstacle zones" within the GI network using Circuitscape and Linkage Mapper software. These analyses identify key areas for optimizing the GI network, providing critical data for guiding improvement efforts.

##### Circuit theory mothed

Circuit theory posits that charges exhibit a property of random wandering^30^. In this framework, landscapes are conceptualized as conductive surfaces, with charges representing ecological flows within complex landscapes. Resistance, analogous to electrical resistance, represents the hindrance to ecological flow diffusion across landscape grids. Resistance values are typically derived from resistance surface grids used in the MCR model^31^. Following the principles of Ohm's law in physics, current in a conductor is directly proportional to the voltage across its ends and inversely proportional to the conductor's resistance. By simulating the wandering behavior of charges in circuits, complex patterns of ecological flow diffusion across landscapes are emulated and analyzed^37^.

##### Centrality degree analysis

Centrality metrics serve to quantitatively analyze the crucial role that Cores and corridors play in the overall connectivity of network systems, with higher node centrality indicating a greater contribution to the network's interconnectedness^27^. The centrality degree measured by Centrality Mapper is simulated by the charge of the ecological flow. The higher the current strength, the stronger the ecological flow of the source, indicating the greater importance of the source^38^. The principle is to input 1A current into an arbitrary hub, and ground the other hubs (arbitrarily), and measure the current strength of each hub in the network.

##### Identification of “PinchPoint” in corridors

“Pinchpoint” is the area where the charge is most concentrated (Or where biological migration density is highest), that is, the area where the ecological flow is concentrated. If this area is destroyed, the ecological flow will be cut off or silted up, which will have a serious impact on the overall connectivity of the network. This study uses the “Pinchpoint” Mapper module in GIS to adopt a many-to-one mode, that is, one hub is grounded, and other hubs input 1A current in turn, and then iterated. Finally, the current value of the grid in the corridor is calculated, and the area with high current is identified as the “pinchpoint”.

##### Identification of “PinchPoint” in corridors

“Pinchpoint” is the area where the charge is most concentrated, that is, the area where the ecological flow is concentrated. If this area is destroyed, the ecological flow will be cut off or silted up, which will have a serious impact on the overall connectivity of the network. This study uses the “Pinchpoint” Mapper module in GIS to adopt a many-to-one mode, that is, one hub is grounded, and other hubs input 1A current in turn, and then iterated. Finally, the current value of the grid in the corridor is calculated, and the area with high current is identified as the “pinchpoint”.

##### Analysis of network barrier

In circuit theory, a “barrier” is an area that strongly impedes the movement of charges. Repairing the “barrier” can significantly improve the connectivity of the network or promote the diffusion process of the ecological flow. The exploration of “barrier” areas is carried out in GIS, and a certain search radius needs to be set. Considering that the GI corridor in urban areas is not too wide, and a search radius of less than 30 m will have a large error^28^, this study sets the search radius to 60 m based on the characteristics of the study area and the grid size. The entire landscape area is searched using the moving window method, and the size of the improvement in overall connectivity after removing a certain area is calculated using the following formula^30,37^:

6 $$\Delta LCD={LCD}_{0}-{LCD}_{1}$$

7 $$IS=\frac{\Delta LCD}{D}$$

where LCD_0_ is the minimum cumulative resistance value in the study area, LCD_1_ is the minimum cumulative resistance value in the study area after removing a certain barrier point, ∆LCD is the decrease in the minimum cumulative resistance value after removing a certain barrier point, D is the search radius in Barrier Mapper, and IS is the improvement value of the connectivity after removing a certain “barrier”.

### Ethical approval and consent to participate

All methods were in compliance with relevant institutional, national, and international guidelines and legislation.

## Results

### Analysis of spatial pattern of GI

**Figure 4:**
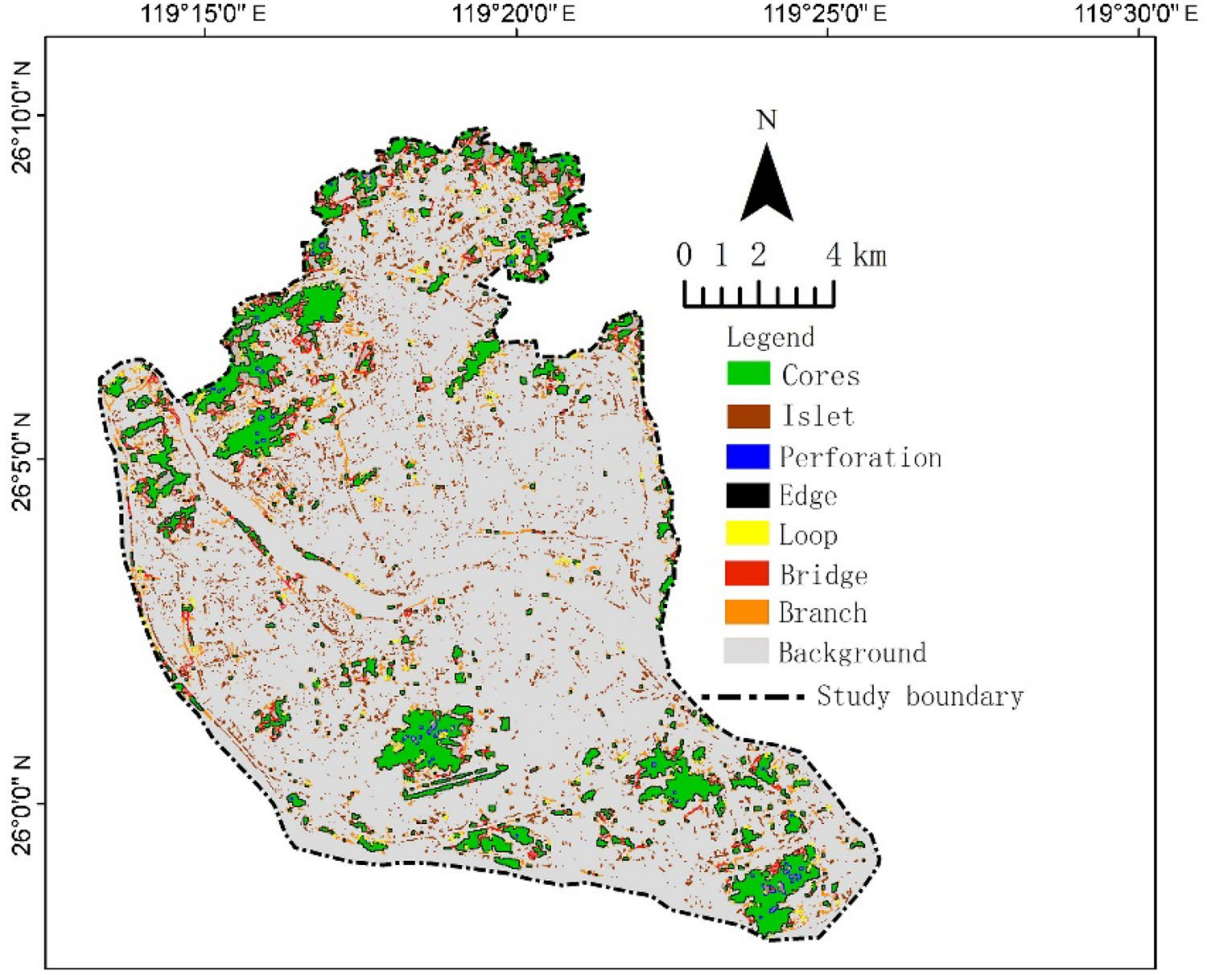
GI spatial structure classification map based on MSPA. Map created in Guidos Toolbox 2.8 and ArcGIS 10.2.

After processing with MSPA, the spatial structure elements and distribution of GI are shown in Fig. 4, and seven types of structural types are statistically analyzed. As displayed in Table 2 and Fig. 4, the total area of GI is 5366.38 ha, accounting for 21.76% of the study area. The majority of GI is distributed in the northwest and south of the study area. The core area of GI has the largest area, totaling 1919.42 ha, accounting for 35.77% of GI and primarily distributed in the urban mountain areas in the north and south of the study area. The bridging area is 401.95 ha, accounting for 7.49% of GI and is more densely distributed in the north than in the south. The edge structure elements cover 1270.4 ha, accounting for 23.67% of GI, which is similar to the distribution of the core area as the edge surrounds the core. The isolated area is 924.19 ha, accounting for 17.22% of GI, and mainly distributed in the main urban area. The perforated area is 55.85 ha, accounting for 1.04% of GI and primarily concentrated inside several larger mountain areas, such as Gaige Mountain, Qingliang mountain, and Meifeng Mountain. The ring road area is 178.17 ha, accounting for 3.32% of GI, while the branch line area is 616.4 ha, accounting for 11.49% of GI.

**Table 2:** Classification and statistical information of GI network spatial structure based on MSPA.

Structural elements	Area (ha)	Quantity	Percentage of total GI area (%)	Percentage of total area (%)
Core	1919.42	625	35.77	7.78
Island	924.19	3245	17.22	3.75
Peforation	55.85	43	1.04	0.23
Edge	1270.40	462	23.67	5.15
Loop	178.17	199	3.32	0.72
Bridge	401.95	400	7.49	1.63
Branch	616.40	2192	11.49	2.50

### Identification of elements of GI network construction

#### Identification of hubs in GI network

**Figure 5:**
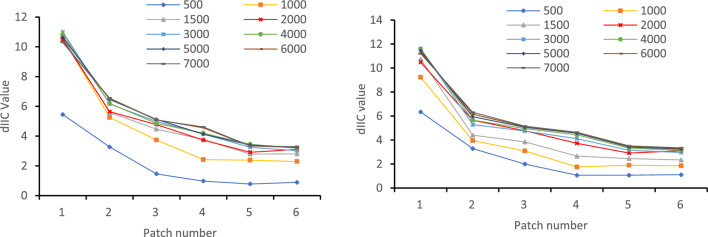
Connectivity trends of patches with distance threshold. Map created in Microsoft Word.

Using nine preset thresholds in Confor 2.6 for test, the change trend of connectivity (dPC and dIIC) is obtained as shown in Fig. 5. The figure shows that dPC and dIIC substantial fluctuations within a distance threshold of 2000 m, and the indicators change less when the distance threshold is above 2000 m, so 2000 m is selected as the distance threshold for analysis.

**Figure 6:**
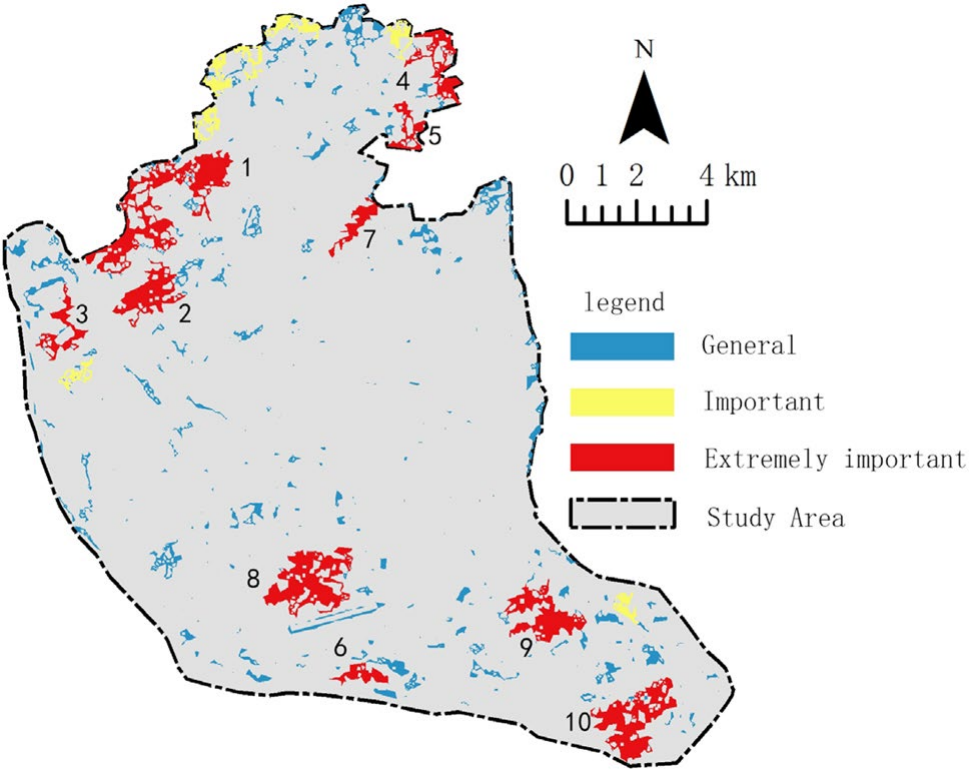
Core, bridge importance evaluation classification. Map created in Guidos Toolbox 2.8 and ArcGIS 10.2.

According to Formula (3), the importance of each patch is calculated, sorted, and graded. Considering practical situations and previous related research, the top 10 patches are selected as hubs as shown in Table 3. Figure 6 shows the grading and spatial distribution of the importance of each patch, with the 10 hubs being extremely important and covering a total area of 1306.98 ha, with 4 in the south and 6 in the north, and none in the central part. The hubs are evidently marginalized and polarized. The important patches are mainly distributed in the edge zone of the north, characterized by good connectivity but small area. There are more patches with moderate importance, but almost none in the middle and east, distributed in other areas, characterized by small area and moderate connectivity.

**Table 3:** The results of source areaimportance index ranking.

Ranking	Lable	dA	dIIC	dPC
1	1	13.666	28.374	30.698
2	8	9.590	18.159	15.486
3	10	8.905	13.935	12.159
4	9	6.384	10.994	10.289
5	2	5.202	10.478	11.354
6	4	3.751	5.642	4.750
7	3	2.669	4.773	4.292
8	7	2.1967	3.728	3.346
9	6	1.802	3.100	2.640
10	5	1.829	2.915	2.788

### Identification of potential GI corridors in Fuzhou

#### Selection of resistance factors and construction of resistance surface

**Figure 7:**
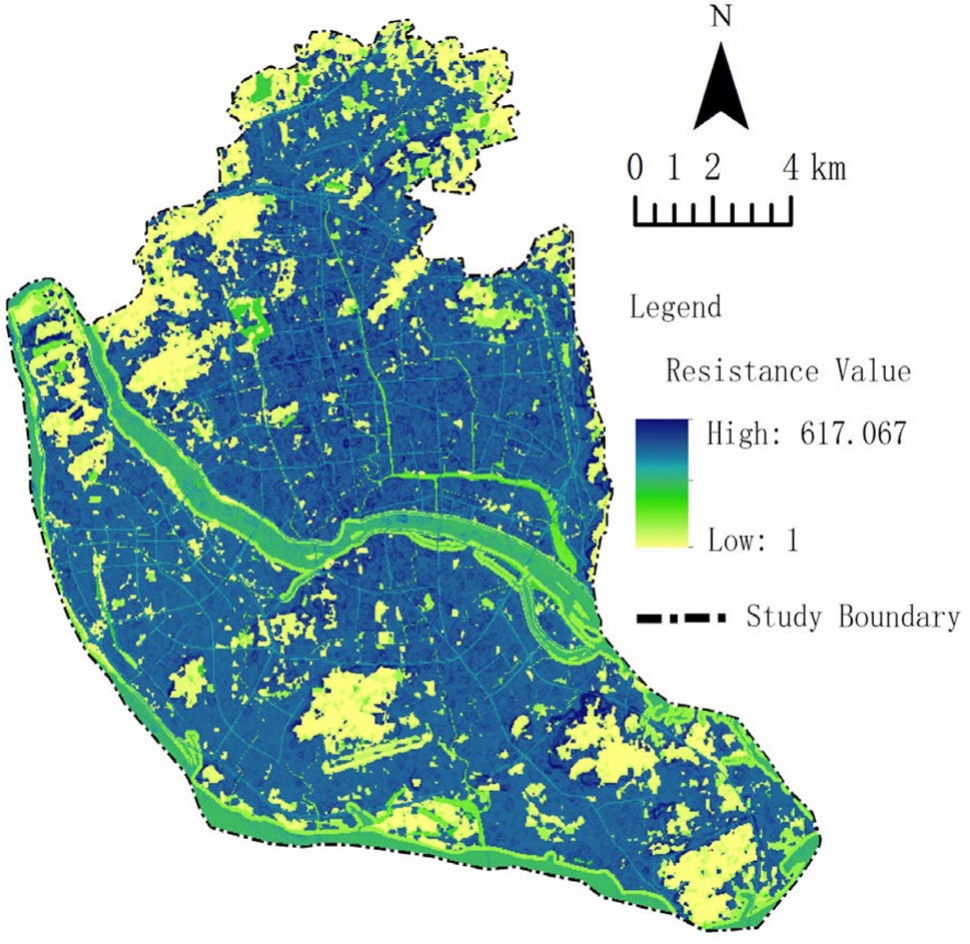
Modified resistance surface. Map created in Guidos ArcGis 10.2.

Through expert consultation and relevant literature^13,32^, land use types and MSPA types were assigned values, and the resistance values for each factor are displayed in Table 4. The slope data of the study area were used for correction to obtain the corrected resistance surface (Fig. 7). The figure shows that the resistance values are unevenly distributed from 1 to 617, with lower resistance primarily in several mountain parks and around water systems, and higher resistance in the urban center.

**Table 4:** Values assigned to ecological resistance factors in Fuzhou city.

Resistance factors	Subcategory	Resistance value	Resistance factors	Subcategory	Resistance value
Hubs	–	1	Water	River	200
Core	Extremely important	1		Lake	150
	Important	10		River and lake 100 m buffer	80
	General	20		Other water bodies	100
Bridge	Extremely important	1	Farmland	–	50
	Important	10	Bareland	–	70
	General	20	road	–	250
Forest	–	30	Construction	–	300

#### Identification of potential corridors

**Figure 8:**
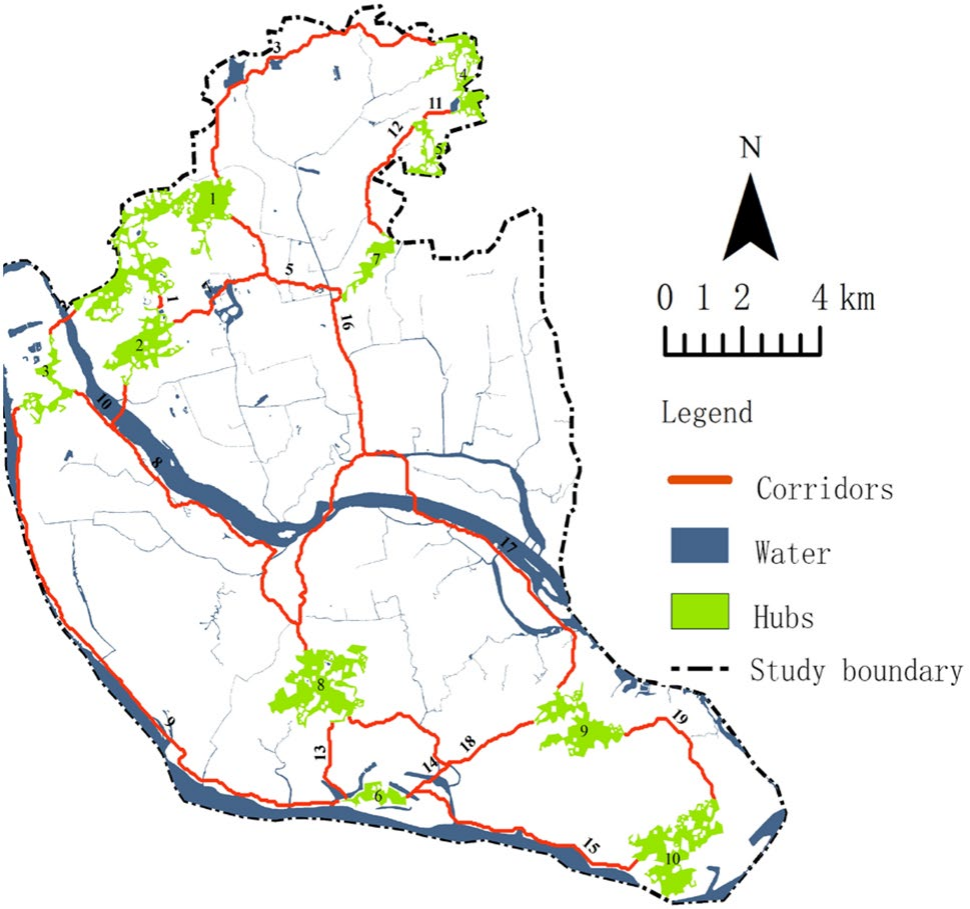
GI network in Fuzhou. Map created in Guidos Circuitscape and ArcGis 10.2.

Based on the corrected resistance surface, the MCR model was used to identify corridors and combined with network hubs to obtain the GI network of Fuzhou (Fig. 8). Among them, there are fewer hubs and corridors in the central area, and most corridors follow the river water system. Table 4 shows that there are 17 GI corridors in the study area, with a total length of 110 km and lengths ranging from 422 m to 15,704 m, with an average length of 6482 m.

Table 5 displays the statistical results of corridor length and resistance. The smaller the cumulative resistance between paired hubs, the higher the possibility of species information exchange between them. The average minimum cumulative resistance value between hubs in the study area was 572,077. The corridor (No. 9) between hub 4 and 5 has the smallest cumulative resistance of 38,418, indicating good accessibility of ecological flow between these two sources. Corridor No. 15 is the longest and has the highest cumulative resistance, passing through the central city area, with a resistance value of 1,417,085. The average resistance value indicates the resistance value per meter of corridor length, and the smaller the average resistance value, the better the ecological conditions along the corridor. Table 4 shows that corridor No. 3 has the lowest average resistance value, with an average value of 37, followed by corridors No. 9 and No. 17, and corridors No. 1 and No. 4 have the highest average resistance value.

**Table 5:** Information statistics of corridor extraction results based on MCR.

Lable	Connected hub	Accumulated resistance	Length of corridor (m)	Average resistance
1	1–2	59,988	422	142
2	1–3	73,281	996	74
3	1–4	343,020	9295	37
4	1–7	560,548	4249	132
5	2–7	625,346	5600	112
6	2–8	865,082	10,428	83
7	3–6	990,067	15,704	63
8	3–8	809,755	10,632	76
9	4–5	38,418	824	47
10	5–7	433,745	3566	122
11	6–8	259,728	2290	113
12	6–9	507,086	4088	124
13	6–10	563,122	7182	78
14	7–8	1,297,007	10,601	122
15	7–9	1,417,085	14,020	101
16	8–9	698,636	6418	109
17	9–10	183,401	3886	47

### Evaluation and optimization of the potential GI network

#### Evaluation of circuit centrality degree and importance classification of GI network hubs

Circuit centrality degree is used to measure the support of the GI network sources in the study area for the network. The higher the centrality degree of the sources, the more potential ecological flow, and the higher the degree of aggregation of species, information, and energy in these hubs, the stronger their role in the network. Table 6 shows that the centrality degree of the 10 hubs ranges from 11.5 to 26.1, with hub 1 and 8 having the highest centrality degrees of 26.1 and 20.2, respectively, indicating that these sources are the most important. Hub 10 has the lowest centrality degree and is located in the southeast corner of the study area, with fewer ecological interactions with other hubs. Hubs 3, 6, 7, and 9 also have relatively high centrality degrees of 17.0, 18.3, 18.6, and 17.9, respectively, indicating frequent material and energy exchange within these sources. Hubs 2, 4, 5, and 10 have lower centrality degrees, indicating lower ecological exchange compared to other hubs. Based on the centrality degree results, the importance of the sources of the GI network in the study area was classified into three levels: high, medium, and low. Overall, based on the centrality degree results, the importance of the GI network sources in the study area was classified into three levels: high, medium, and low. Hubs 1 and 8 are considered extremely important, hubs 3, 6, 7, and 9 are relatively important, and hubs 2, 4, 5, and 10 are generally important.

**Table 6:** Quantitative analysis of electric circuit centrality of GI network hub in Fuzhou.

Hub labe	Centrality
1	26.1
2	15.6
3	18.3
4	15.7
5	14.4
6	17.9
7	18.6
8	20.2
9	17.0
10	11.5

### Identification and protection of circuit “Pinchpoint” in the GI network

**Figure 9:**
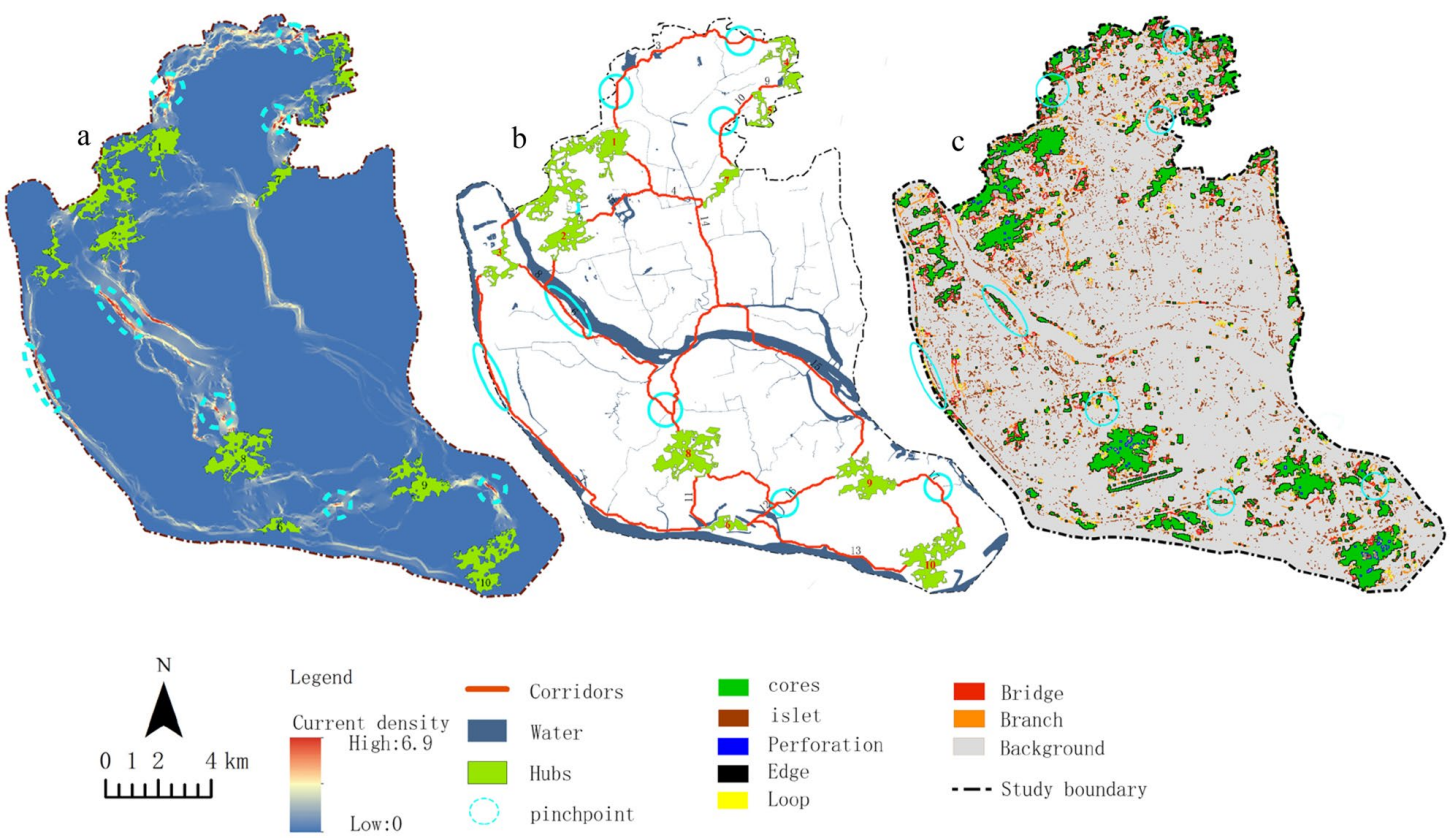
Distribution of Pinchpoint in the GI network of Fuzhou. Map created in Guidos Circuitscape and ArcGis 10.2.

According to circuit theory, the “Pinchpoint” in the GI network play an essential role in maintaining the overall connectivity and ecological flow of the region. Identifying and protecting these “Pinchpoint” can effectively enhance the ecological function of the GI network. Figure 9a shows that there are nine “Pinchpoint” identified in the GI network of the study area, primarily distributed in the north–south regions of Cangshan and Jin’an, with fewer in Gulou and Taijiang. Figure 9b shows the locations of the nine circuit “Pinchpoint” in the GI network corridors, which are the areas that need the most attention and protection in GI network construction. Not all corridors have “Pinchpoint”, such as corridors 4, 5, 11, 13, and 15. Figure 9c shows the MSPA element types where the “Pinchpoint” are located, with most corresponding to core elements, followed by bridging and branch elements, and background elements having almost no “Pinchpoint”.

### Detection of “Barrier” areas in the GI network

**Figure 10:**
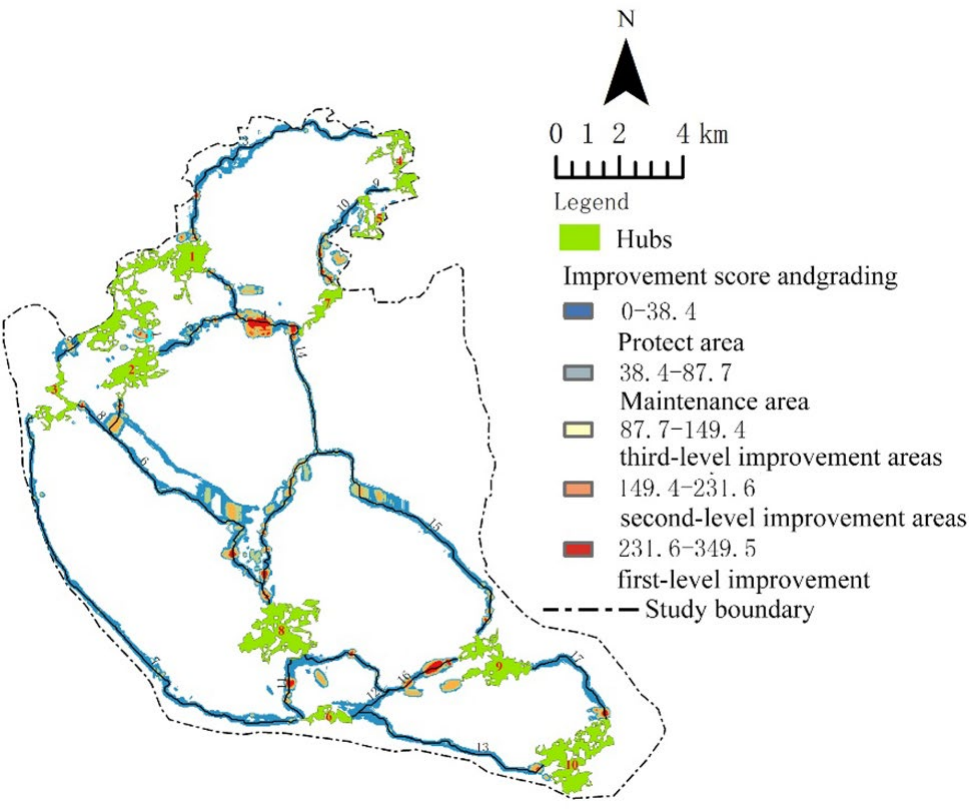
illustrates the distribution of “Barrier” within the green infrastructure network of Fuzhou city. Map created in Guidos Circuitscape and ArcGis 10.2.

According to circuit theory, “Barrier” areas refer to areas with less charge distribution, that is, areas where ecological flow is obstructed in the GI network and require ecological improvement. Figure 10 shows the identification results of “Barrier” areas in the GI network of the study area, divided into four levels based on the possibility of improvement using the natural break method: nonbarrier areas with low values (below 87.7), and first, second, and third-level improvement areas with values above 87.7, totaling 595.9 ha. Figure 10 shows that there are 68 first-level improvement areas, with a total area of 73.59 ha, accounting for 12.4% of the improvement areas, mainly distributed around hub 8, followed by hub 7. There are 149 s-level improvement areas, with an area of 203.4 ha, accounting for 34.1% of the improvement areas, mainly distributed in corridor 14, followed by corridors 10 and 15. There are 463 third-level improvement areas, with a total area of 319 ha, accounting for 53.5% of the improvement areas. Third-level improvement areas are mainly distributed in corridors 14 and 15, with the exception of hub 9, and are present in all other corridors.

## Discussion

### Revealing the spatial pattern of GI in Fuzhou City

This study focuses on the main urban areas of cities, revealing that the total amount of land allocated for GI within these areas is insufficient and unevenly distributed, particularly scarce in high-density urban districts. This finding aligns with the research conducted by Yao Xiong et al.^39^. In similar studies by Geng et al.^40^, Jia et al.^41^, and colleagues, a higher proportion of GI land use was observed, attributed to the inclusion of surrounding rural and township lands, which feature more extensive forested areas.

The total area of GI in the study area is relatively small, particularly in the central region. The core areas of GI serve as crucial habitats for regional species, and they exhibit clear north–south differentiation within the study area. Strengthening the connection and communication between the two regions is necessary. The primary reason for the northward concentration of the core area is its location on the city’s periphery, with relatively less human interference. The southward concentration is due to the lower level of development and construction in the relatively late-developed Cangshan district. In contrast, the central region lacks core areas and has numerous isolated GI, mainly due to it being the area with the highest population density and construction density in Fuzhou. Large Greenland has been encroached upon, leaving behind isolated GI. In future construction, the GI in the central region should be strengthened, such as by relying on water systems, cultural heritage sites, and increasing green space. Additionally, the proportion of core areas in the study area is not high, indicating that the fragmentation of GI in the study area is severe and requires increased GI construction. The distribution of bridging structures, which connect different core areas of GI, exhibits polarization along the north–south axis. This indicates that the connectivity of GI is relatively good between the north and south, whereas the connection between the central region and the north and south needs to be achieved by building bridges and corridors.

### Identifying the GI network in Fuzhou City

#### Hubs of the GI network in Fuzhou City

The identification of hubs in the GI network in Fuzhou City was based on source-site importance evaluation, which includes biomass and the potential for species migration and diffusion^4,20^. Previous studies have only considered the spatial connectivity level of the source site itself^23,24^, but the ecological conditions provided by the source site have not been adequately considered. In this study, the importance of the source site was quantitatively described by the area indicator, connectivity probability, and overall connectivity index, based on the extraction of bridging and core elements using MSPA. Then, they were ranked and classified to select network hubs. We found that the most important and significant patches of GI in the study area were primarily distributed in the north, indicating that the quality of the patches in this area was generally better. These patches had good connectivity, and the possibility of material and energy interaction was relatively high. In terms of importance distribution, the quality of GI patches in the north was the best, followed by the south, and the central region was the worst, which was directly related to the strength of human activities because the main urban area of the study area is located in the central region. From the distribution of hubs, there were no hubs in the central region. The corridor between the GI in the north and south will be relatively long and not conducive to the interaction of ecological flows. Therefore, ecological construction in the central region should be increased in future construction, and hubs can be added to the GI network to connect the north and south GI.

#### Identification of potential GI corridors in Fuzhou City

The characteristics of GI corridors significantly impact the ecological flow between network hubs. The length and resistance of corridors directly affect the possibility of material, energy, and other information exchange. If the resistance of a corridor is too high, such as in large areas of rivers or buildings, it will impede the flow of ecological resources. Moreover, if the corridor is too long, it is not conducive to the migration and diffusion of certain species^42–44^. The extraction results of GI corridors in the study area showed that the corridors in the north were superior to those in the south (in terms of quantity and average length), indicating better connectivity of patches in the north. However, the corridors that connect the north and south are relatively long (averaging 12.3 km), which is not conducive to species migration and diffusion. Therefore, hubs should be added in the middle of the corridors in future construction. The addition of hubs can be appropriately set according to distance or weighted resistance to promote ecological flow interaction. In addition, if the resistance in the corridor is too high, it will also affect the ecological flow interaction. For example, the first corridor has a short length (422 m), but the average resistance is high, reaching 142. The reason for this is that the corridor passes through construction land. We suggest increasing ecological construction on this corridor to improve the GI network.

This research primarily investigates urban areas situated in high-density mountainous terrains, resulting in a relatively lower number and area of nodes, leading to a more fragmented urban landscape. Similar studies conducted by Huang et al.^26^ in these regions selected 13 nodes with a similar distribution pattern. However, due to their larger scope and the inclusion of extensive forest lands, not only were the number and area of nodes larger, but the average area per node was also greater. However, their selection of nodes, based solely on connectivity without considering area, overlooked relatively isolated and larger green spaces within cities, which play a crucial role in the ecological health of urban regions. Excluding these green areas represents a significant loss. In this study, isolated urban green patches are also connected to surrounding Hubs, forming a network beneficial to the internal ecosystem's health. The identification of corridors in this study utilizes the MCR model, which requires the resistance surface of the study area as foundational data. As the study area encompasses mountainous urban regions, this research adapts the basic resistance surface with topographic factors, aligning more closely with the actual conditions of mountainous terrain. Previous studies by Huang et al.^26^, Li et al.^45^, and others on constructing networks in mountainous terrains have shown inadequacies in considering topography for corridor selection, leading to less scientific choices in areas with significant elevation changes. The subjectivity in assigning resistance factors and their values is commonly strong in current related research^24,25,46^, suggesting a need for further exploration into the impact of different resistance factors and valuation methods on corridor selection, to establish a more scientific and objective basis for constructing resistance surfaces.

### Optimizing the GI network in Fuzhou City

#### Protection and optimization of GI network hubs in Fuzhou City

Based on the centrality of hubs, the protection of GI network hubs in Fuzhou City can be divided into three levels^38^. The most important hubs for protection are hubs 1 and 8, the relatively important hubs are hubs 3, 6, 7, and 9, and the generally important hubs are hubs 2, 4, 5, and 10. Hubs 1 and 8 have the highest centrality and larger areas, and are surrounded by the most other GI network sources. Hub 8 also has the most connections to GI network corridors. Therefore, they play a significant role in supporting the overall GI network, both in terms of ecological benefits and connectivity, making them suitable for being designated as extremely important protected areas. Relatively important protected hubs, such as hubs 7 and 9, are more distributed within Fuzhou City and are surrounded by more hubs. They play an important role in transmitting ecological services provided by the GI network to the urban area and provide a basis for the expansion of the GI network within the city. They also have an important role in connectivity. Generally important protected hubs are more edge-distributed, not only serving as important hubs in the main urban area of Fuzhou City’s GI network, but also providing a platform and possibility for more open exchange with the outside world. They are an indispensable part of Fuzhou City’s GI network and should be protected accordingly.

#### Optimization of potential GI network corridors in Fuzhou City

In this study, we employed the “Pinchpoint” and “barrier” detection method from electrical circuit theory to identify eight key areas for protection and three levels of improvement zones in the corridors^47^. The “Pinchpoint” in the GI network is the most concentrated area of ecological flow. These eight “Pinchpoint” areas are not only crucial components of the GI network but also play a critical role in maintaining the connectivity of the entire network^28^. Therefore, they should be considered as key areas for protection. The term “Barrier” zone denotes areas that markedly impede the transmission of ecological flow. Ecological development strategies can be implemented in these regions, which encompass the expansion of green spaces and the enhancement of their quality. The purpose of this approach is to mitigate barriers within these “Barrier” zones, thus effectively augmenting network connectivity through the elimination of such restrictions^15^. Based on the “barrier” value, the corridors in the study area were divided into three levels of improvable and maintainable areas, with the 68 first-level improvement zones having the highest improvement values. Removing these barriers (i.e., improving the ecological conditions in these areas) will strongly promote the overall connectivity of the GI network in the study area, making it the area with the greatest need for improvement^48^. Whether it is a first-level or second- or third-level improvement area, their ecological construction will benefit the optimization of the Fuzhou GI network. The nonbarrier areas are regions of the network that require little or no improvement. For example, according to the detection results, the entire 9th corridor is a non-barrier area, indicating that the current conditions of this corridor in the GI network in the study area are the best, and the focus should be on its protection in the planning and management of the GI network.

This study employs circuit theory to optimize the GI network in Fuzhou City, conducting a scientific assessment of the magnitude and resistance of ecological flows between nodes and corridors. Compared to traditional network structure analysis methods based on graph theory, this approach offers greater practicality and scientific validity. Unlike the network structure evaluation methods used by Xu et al.^24^, Li Hua et al.^45^, which enhance the network by increasing corridor connectivity and closure through nodes, this method not only evaluates the magnitude of ecological flows and the ecological processes between nodes but also quantitatively assesses the ecological condition of existing corridors. This includes identifying "pinch points" and "barriers" within corridors, thus providing more guidance on how to improve current nodes and corridors.

## Limitations and future work

Although we have employed a series of methods and measures to identify and optimize the GI network in Fuzhou City, offering insights for related research and construction in mountainous urban areas, we acknowledge certain limitations and areas for improvement in our process. Firstly, in the selection of GI network nodes, the use of land area as a proxy for quality lacks comprehensiveness. While Fuzhou's location in the South Asian maritime monsoon climate zone means that green spaces are predominantly arboreal and shrubby, and the size of an area can to some extent represent the quality of green land, a more precise identification of GI network nodes necessitates further investigation and evaluation of GI elements. Secondly, in constructing the resistance surface, despite our review of extensive literature and adoption of expert assessment to select appropriate factors and assign objective values, the corridor routing remains influenced by the magnitude of resistance values and the gradient effect, leading to potential deviations in corridor positioning and routing. A possible solution is to conduct long-term observations and studies on specific species, especially regional keystone species, as selecting corridors based on these species would offer greater practical relevance. Lastly, our research also encounters issues related to scale or parameters, such as a grid size of 30 m, under which smaller green spaces might be overlooked. In the MSPA, settings such as edge width and neighborhood rules, determined through literature review, impact the quantity of MSPA type elements within the GI network. For instance, increasing the edge width parameter would decrease the number and area of Cores while enlarging the Edge area.

This study employed methods such as MSPA, MCR, and circuit theory to construct and optimize the GI network, offering valuable insights for the planning of GI, ecological planning, and sustainable strategies in modern urban settings. Particularly for mountainous cities, this research introduces a method for constructing resistance surfaces adjusted for topography, ensuring that corridor selection considers topographical factors more objectively. By considering green spaces of a certain area as nodes within urban regions, the connectivity of the city's GI is enhanced, simultaneously improving the ecological quality of high-density urban areas. Furthermore, the application of circuit theory for optimizing the GI network not only facilitates the assessment of current nodes and corridors but also thoroughly accounts for ecological processes, clearly identifying areas of the network with good and poor ecological quality. This provides strong practical guidance for related network optimization research and practice. Overall, this paper proposes a method for ecological planning of GI networks in mountainous cities that is straightforward, accessible, and reliable for data collection, suitable for long-term monitoring, and applicable to the ecological management and adjustment of densely populated and economically advanced mountainous urban areas, offering a pathway for regional planning.

## Conclusion

This research targets the main urban area of Fuzhou City, employing methods such as the MSPA, area and connectivity indices, the MCR model, and circuit theory to construct and optimize the GI network. The MSPA reveals the spatial pattern of Fuzhou City's GI, significantly informing the construction and development of regional GI networks. The use of area and connectivity indices to identify crucial source lands as GI network Hubs ensures that the selection considers both the quality of the source lands and the importance of spatial patterns, enhancing the reliability of the results. The adoption of resistance surfaces adjusted for topographic slope and the MCR model to extract GI network corridors yields results that are more aligned with the actual conditions of mountainous urban areas. Lastly, the application of circuit theory to evaluate and optimize the GI network utilizes the model of electrical charges moving freely across resistance surfaces to simulate the migration and dispersion of species. This approach, compared to other network structure evaluation and optimization methods, more accurately represents ecological processes. Based on the circuit theory, this study proposes targeted areas for protection and enhanced construction within the GI network, highlighting critical zones needing preservation and weaker areas requiring strengthening, offering new pathways for GI network optimization.

## Data Availability

The datasets used during the current study are available from the corresponding author on reasonable request.
